# Supplementary material for: Structural analysis of full-length SARS-CoV-2 spike protein from an advanced vaccine candidate
Source: bioRxiv. 2020 Aug 6:2020.08.06.234674. Preprint. [Version 1] doi: 10.1101/2020.08.06.234674 (PMC7418715; doi:10.1101/2020.08.06.234674)
Supplement: 1 [file NIHPP2020.08.06.234674-supplement-1.pdf]

bioRxiv preprint doi: https://doi.org/10.1101/2020.08.06.234674. this version posted August 6, 2020. The copyright holder for this preprint (which was not certified by peer review) is the author/funder. It is made available under a CC-BY 4.0 International license.

Supplementary Materials for

Structural analysis of full-length SARS-CoV-2 spike protein from an advanced vaccine candidate

Sandhya Bangaru[1], Gabriel Ozorowski[1], Hannah L. Turner[1], Aleksandar Antanasijevic[1], Deli Huang[2], Xiaoning Wang[3], Jonathan L. Torres[1], Jolene K. Diedrich[3], Jing-Hui Tian[4], Alyse D. Portnoff[4], Nita Patel[4], Michael J. Massare[4], John R. Yates III[3], David Nemazee[2], James C. Paulson[2,3], Greg Glenn[4], Gale Smith[4] and Andrew B. Ward[1*]

Correspondence to: andrew@scripps.edu

This PDF file includes:

Materials and Methods
Supplementary Text
Figs. S1 to S4
Table S1

bioRxiv preprint doi: https://doi.org/10.1101/2020.08.06.234674. this version posted August 6, 2020. The copyright holder for this preprint (which was not certified by peer review) is the author/funder. It is made available under a CC-BY 4.0 International license.

SUPPLEMENTARY MATERIALS

Materials and Methods

Recombinant full-length SARS-CoV-2 prefusion S. SARS-CoV-2 full-length constructs were synthesized from the S glycoprotein gene sequence (GenBank MN908947 nucleotides 21563-25384). The wild-type full-length gene was codon optimized for expression in *Spodoptera frugiperda* (Sf9) cells by GenScript (Piscataway, NJ, USA). Amino acid mutations in the S1/S2 cleavage domain were introduced in the furin cleavage site (RRAR to QQAQ) to be protease resistant (SARS-CoV-2 3Q S) and in a second construct two proline mutations (K986P and V987P) were introduced in the HR1 domain (SARS-CoV-2 3Q-2P S) to stabilize the envelope proteins in a prefusion conformation (*18*).

Expression and purification. For expression of SARS-CoV-2 full-length spike (S) proteins, the synthetic S-genes were codon optimized for insect cells, cloned into the pBac1 baculovirus transfer vector (Millipore, Sigma), and co-transfected into *S. frugiperda* [Lepidoptera] Sf9 cells with the flashBACTM GOLD system (Oxford Expression Technologies) using X-tremeGENE HP transfection reagent (Roche). Recombinant baculovirus-infected cells were harvested by centrifugation, SARS-CoV-2 S envelope proteins extracted with non-ionic detergent and purified using anion exchange and lentil lectin affinity column chromatography. Purified SARS-CoV-2 3Q and 3Q-2P S proteins were dialyzed in 25 mM sodium phosphate (pH 7.2), 300 mM NaCl, 0.01% (v/v) polysorbate 80 (PS 80) and stored at -80$_o$ C.

bioRxiv preprint doi: https://doi.org/10.1101/2020.08.06.234674. this version posted August 6, 2020. The copyright holder for this preprint (which was not certified by peer review) is the author/funder. It is made available under a CC-BY 4.0 International license.

Ns-EM sample preparation and data collection. Equal concentrations of SARS-CoV-2-3Q-2P full-length spike formulated in PS80 and Matrix adjuvant were diluted to approximately 20 µg/mL with TBS. The sample was directly deposited onto carbon-coated 400-mesh copper grids and stained immediately with 2% (w/v) uranyl formate for 90 seconds. Grids were imaged at 120 KeV on Tecnai T12 Spirit with a 4k x 4k Eagle CCD camera at 52,000x magnification and -1.5 µm nominal defocus. Micrographs were collected using Leginon and the images were transferred to Appion for processing (*41, 42*). Particle stacks were generated in Appion with particles picked using a difference-of-Gaussians picker (DoG-picker) and 2D classes generated by MSA/MRA (*43, 44*).

Cryo-EM sample preparation. For SARS-CoV-2 3Q-2P, 3.5 µL of spike at 0.4 mg/mL was mixed with 0.5 µL of 0.04 mM lauryl maltose neopentyl glycol (LMNG) solution immediately before sample deposition onto a 1.2/1.3 300-Gold grid (EMS) that had been plasma cleaned for 7 seconds using a Gatan Solarus 950 Plasma system. Following sample application, grids were blotted for 3 seconds before being vitrified in liquid ethane using a Vitrobot Mark IV (Thermo Fisher). For SARS-CoV-2 3Q, 3.5 µL of spike at 0.5 mg/mL was mixed with 0.5 µL of 0.04 mM of LMNG and frozen in a similar manner.

Cryo-EM data collection and processing. For collection, a Talos Arctica TEM at 200 kV was used with a Gatan K2 Summit detector at a magnification of 36,000x, resulting in a 1.15 Å pixel size. Total exposure was split into 250 ms frames with a total cumulative dose of ~50 $e^-/Å^2$. Micrographs were collected through Leginon software at a nominal defocus range of -0.5 µm to -1.6 µm for 3Q-2P spike and at a defocus range of -0.4 µm

bioRxiv preprint doi: https://doi.org/10.1101/2020.08.06.234674. this version posted August 6, 2020. The copyright holder for this preprint (which was not certified by peer review) is the author/funder. It is made available under a CC-BY 4.0 International license.

to -2.3 μm for 3Q spike (*45*). MotionCor2 was used for alignment and dose weighting of the frames (*46*). Micrographs were transferred to CryoSPARC 2.9 for further processing (*47*). CTF estimations were performed using GCTF and micrographs were selected using the Curate Exposures tool in CryoSPARC based on their CTF resolution estimates (cutoff 5 Å) for downstream particle picking, extraction and iterative rounds of 2D classification and selection (*48*). Particles selected from 2D classes were used for 3D refinement of free trimers for 3Q-2P-FL and 3Q-FL datasets in CryoSPARC. Final subsets of clean trimer particles were refined with C3 symmetry and local resolution for the free trimer was calculated using the local resolution function in CryoSPARC. Particles corresponding to dimers-of-trimers classes in CryoSPARC were transferred to Relion 3.0 for iterative rounds of 3D classification to separate dimers-of-trimers and trimers-of-trimers (*49*). Final subsets of clean particles from dimers-of-trimers class were refined with C2 symmetry and the trimers-of-trimers class with C1 symmetry.

Model building and refinement. The 3.6 Å C3-symmetric free trimer map and the 4.5 Å C2-symmetric dimers-of-trimers maps were used for model building and refinement. Initial model building was performed manually in Coot using PDB 6VXX as a template followed by iterative rounds of Rosetta relaxed refinement and Coot manual refinement to generate the final models (*50, 51*). EMRinger and MolProbity were run following each round of Rosetta refinement to evaluate and choose the best refined models (*52, 53*). To prepare linoleic acid and PS 80 detergent ligands for modeling, PDB and CIF ligand definition files were created using Phenix eLBOW (*54*) by providing the SMILES string for PubChem CID: 5284448 (polysorbate 80) or the PDB chemical component code EIC (linoleic acid).

bioRxiv preprint doi: https://doi.org/10.1101/2020.08.06.234674. this version posted August 6, 2020. The copyright holder for this preprint (which was not certified by peer review) is the author/funder. It is made available under a CC-BY 4.0 International license.

The coordinates were manually placed and refined into the respective map densities using Coot. For Rosetta refinement, each ligand was saved in MOL2 format and Rosetta parameter files were generated using the molfile_to_params.py function (*51*). Final map and model statistics are summarized in Table S1. Figures were generated using UCSF Chimera and UCSF Chimera X (*55, 56*).

Mass-spectrometry. Mass spectrometry to identify fatty acids in the SARS-3Q-2P-FL protein was performed as described previously (*21*). We obtained several candidates in this screen that were narrowed down to 6 candidates based on their intensity and the m/z range of 250-300.

Sequence alignment. The sequences for the analysis were obtained either from GISAID or NCBI GenBank. GISAID accession number or the GenBank accession id from which whole genome sequences were obtained are as follows: Bat-SL-RatG13 (EPI_ISL_402131), Bat-SL-CoVZC45 (MG772933.1), BetaCoV/pangolin/Guandong/1/2019 (EPI_ISL_410721) and BetaCoV/pangolin/Guangxi/P4L/2007 (EPI_ISL_410538), SARS-CoV Tor2 strain (NC_004718.3). The spike sequences were extracted and aligned using Clustal Omega (*57*).

Pseudovirus (PSV) assay. Pseudovirus preparation and assay were performed as previously described (*58*). Under BSL2/3 conditions, MLV-gag/pol and MLV-CMV plasmids was co-transfected into HEK293T cells along with full-length or mutant SARS-

bioRxiv preprint doi: https://doi.org/10.1101/2020.08.06.234674. this version posted August 6, 2020. The copyright holder for this preprint (which was not certified by peer review) is the author/funder. It is made available under a CC-BY 4.0 International license.

CoV-2 spike plasmids using Lipofectamine 2000 to produce a single round of infection competent pseudo-viruses. The medium was changed 12 hours after transfection. Supernatants containing the viruses were harvested 48h after transfection. In sterile 96-well half-area plates, 25 µl of virus was immediately added to 10,000 HeLa or HeLa-ACE2 cells in 75 µl of medium. Plates were incubated at 37°C for 42 to 48 h. Following the infection, HeLa and HeLa-hACE2 cells were lysed using 1x luciferase lysis buffer (25 mM Gly-Gly pH 7.8, 15 mM $MgSO_4$, 4 mM EGTA, 1% Triton X-100). Luciferase intensity was then read on a Luminometer with luciferase substrate according to the manufacturer's instructions (Promega, PR-E2620).

Site-specific glycosylation

A sample of the SARS-CoV-2 prefusion spike protein expressed in the SF9 insect cell line was prepared for MS analysis as previously described with minor modifications (*24*). In brief, the protein (50 µg) was denatured and aliquots (10 µg) were digested under five different protease conditions including chymotrypsin, a combination of trypsin and chymotrypsin, trypsin, elastase and subtilisin as described. All samples were then pooled and deglycosylated by Endo H followed by PNGase F in O_{18}-water. To obtain full site coverage, an additional aliquot of the denatured protein (10 µg) sample was digested with chymotrypsin (1:13 w/w) only and deglycosylated with EndoH and PNGase F like the other samples.

The combined protease-treated and chymotrypsin only samples were separately analyzed on an Q Exactive HF-X mass spectrometer (Thermo). Each sample was run twice as replicate. Samples were injected directly onto a 25 cm, 100 µm ID column packed

bioRxiv preprint doi: https://doi.org/10.1101/2020.08.06.234674. this version posted August 6, 2020. The copyright holder for this preprint (which was not certified by peer review) is the author/funder. It is made available under a CC-BY 4.0 International license.

with BEH 1.7 μm C18 resin (Waters). Samples were separated at a flow rate of 300 nL/min on a nLC 1200 (Thermo). Solutions A and B were 0.1% formic acid in 5% and 80% acetonitrile, respectively. A gradient of 1–25% B over 160 min, an increase to 40% B over 40 min, an increase to 90% B over another 10 min and held at 90% B for 30 min was used for a 240 min total run time. Column was re-equilibrated with solution A prior to the injection of sample. Peptides were eluted directly from the tip of the column and nanosprayed directly into the mass spectrometer by application of 2.8 kV voltage at the back of the column. The HFX was operated in a data dependent mode. Full MS1 scans were collected in the Orbitrap at 120k resolution. The ten most abundant ions per scan were selected for HCD MS/MS at 25NCE. Dynamic exclusion was enabled with exclusion duration of 10 s and singly charged ions were excluded.

The MS data were processed essentially as described previously (*24*). The data were searched against the proteome database and quantified using peak area in Integrated Proteomics Pipeline-IP2. Since the processing pathway in SF9 cell line (insect cell line) is similar to mammalian cells for oligomannose and hybrid structures cleaved by Endo-H, and then diverges to produce a combination of paucimannose and complex type glycans, peptides with N+203 were identified as having oligomannose type glycans, and peptides with N+3 are assigned as peptides with complex and paucimannose type glycans.

bioRxiv preprint doi: https://doi.org/10.1101/2020.08.06.234674. this version posted August 6, 2020. The copyright holder for this preprint (which was not certified by peer review) is the author/funder. It is made available under a CC-BY 4.0 International license.

Figures

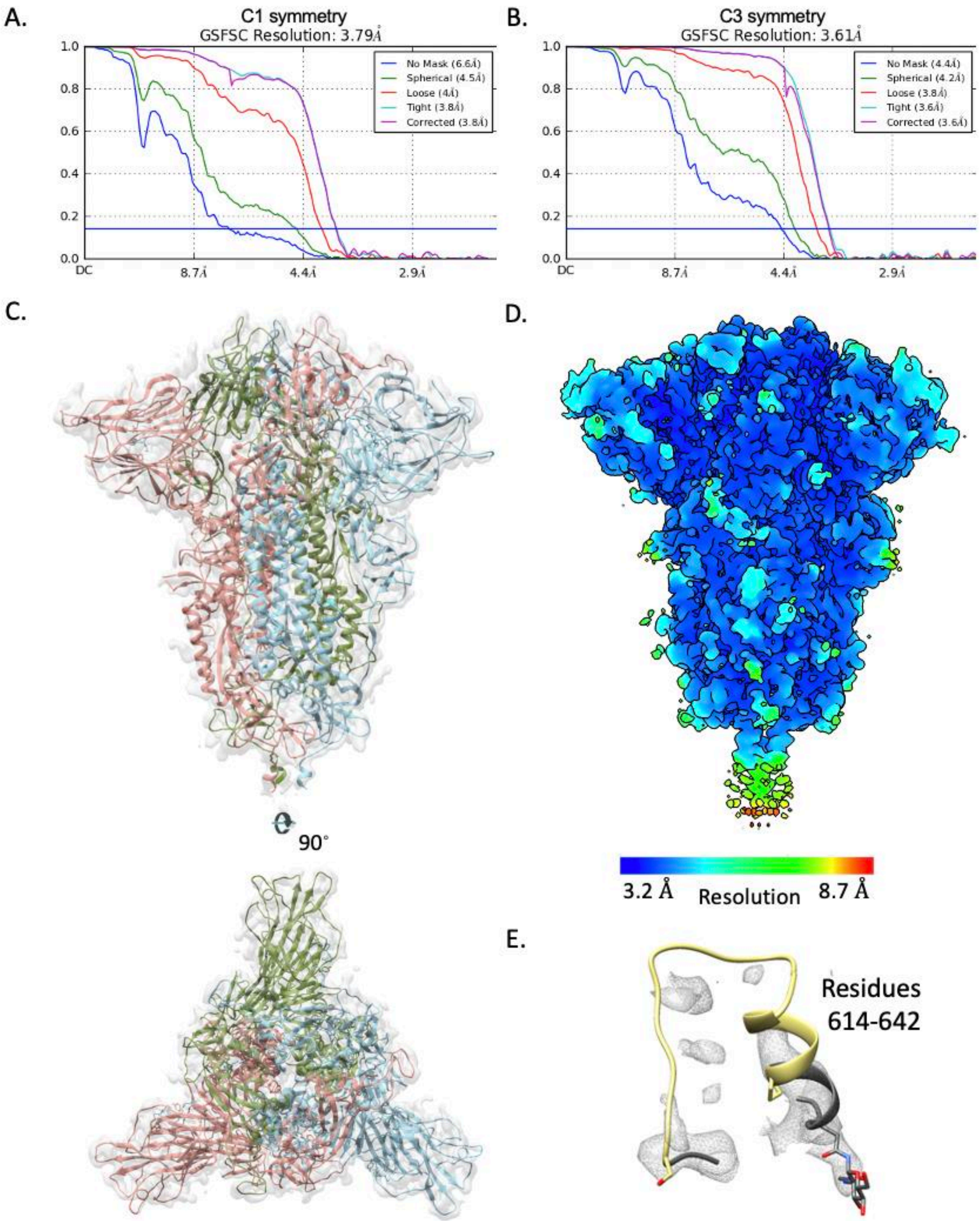

Figure S1. Cryo-EM structure analysis and validation of SARS-CoV-2 3Q-2P-FL spike trimer. (A) FSC curve for SARS-CoV-2 3Q-2P-FL spike when C1 symmetry was imposed during refinement. **(B)** FSC curve for SARS-CoV-2 3Q-2P-FL spike with C3 symmetry imposed. **(C)** Side and top view of the C3 trimer atomic model represented as a ribbon diagram fit into the 3Q-2P-FL spike C1 map density. The protomers are colored

bioRxiv preprint doi: https://doi.org/10.1101/2020.08.06.234674. this version posted August 6, 2020. The copyright holder for this preprint (which was not certified by peer review) is the author/funder. It is made available under a CC-BY 4.0 International license.

in blue, green and coral and the map is shown as a transparent gray density. **(D)** Cryo-EM map of SARS-CoV-2 3Q-2P-FL C3, colored according to local resolution. **(E)** Ribbon representation of residues 614-642 of the PDB model 6X6P with their corresponding cryo-EM density (EMD-22078) shown as mesh representation. Residues P621 to S640 are colored in yellow.

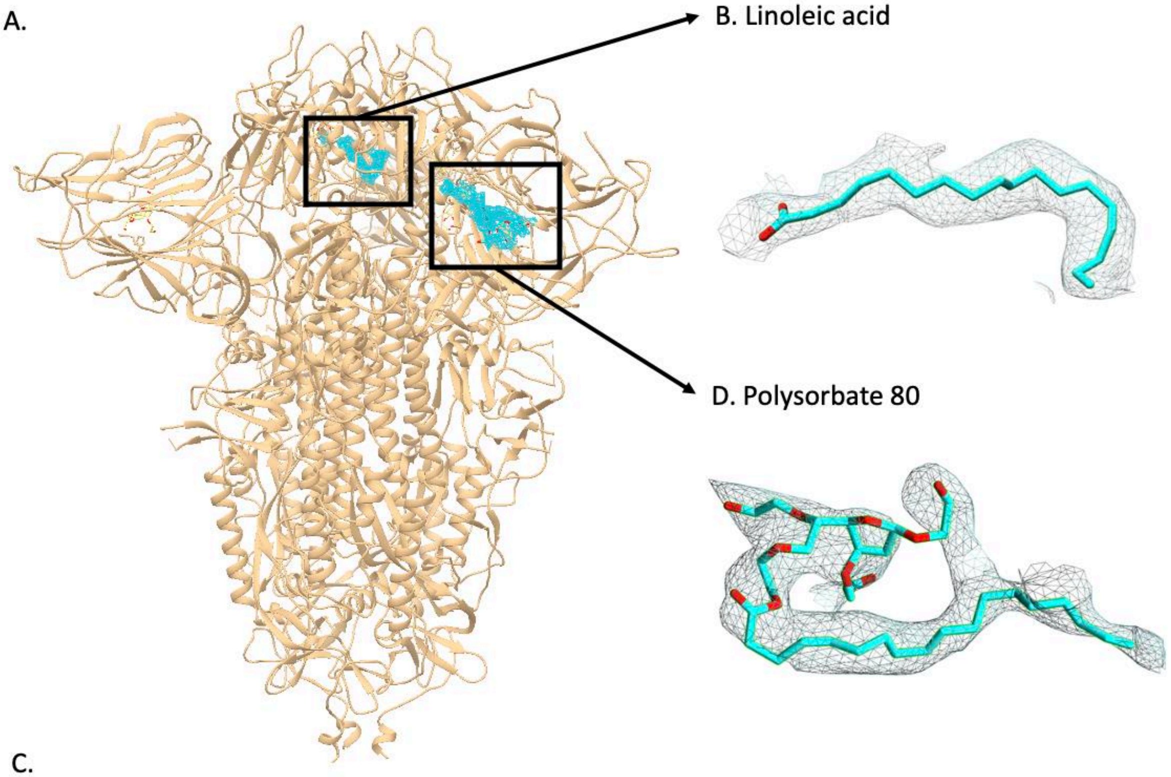

C.

mz	Max intensity	possible candidates
257.25	236774	Palmitic acid; Isopalmitic acid
271.23	42960	Ambrettolic acid
281.25	113796	**Linoleic acid**; Stearolic acid
283.26	4387786	Oleic Acid; Elaidic Acid; Vaccenic acid
283.26	38888	Oleic Acid; Elaidic Acid; Vaccenic acid
285.28	124248	Stearic acid

Figure S2. Ligands found in the EM map of the SARS-CoV-2 3Q-2P-FL spike trimer. (A) Side view of the trimer atomic model represented as a ribbon diagram in tan with the two ligands and their corresponding map densities shown as mesh in cyan. **(B)** Linoleic acid and its corresponding map density shown as mesh in cyan. **(C)** The potential ligand candidates obtained from the mass spectrometry analysis of the SARS-CoV-2 3Q-2P-FL spike trimer with their corresponding size and signal intensity. **(D)** Polysorbate 80 and its corresponding map density shown as mesh in cyan.

bioRxiv preprint doi: https://doi.org/10.1101/2020.08.06.234674. this version posted August 6, 2020. The copyright holder for this preprint (which was not certified by peer review) is the author/funder. It is made available under a CC-BY 4.0 International license.

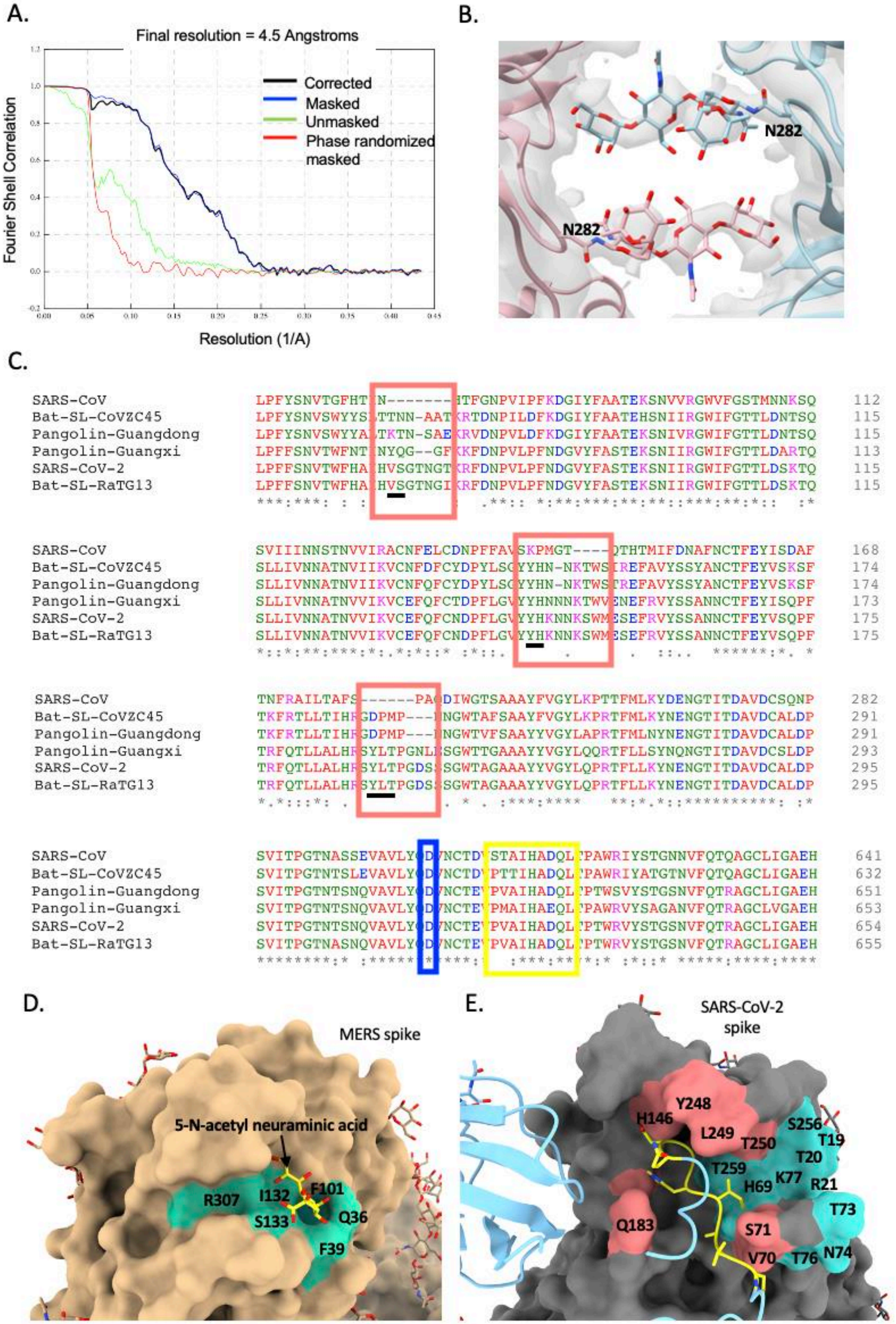

bioRxiv preprint doi: https://doi.org/10.1101/2020.08.06.234674. this version posted August 6, 2020. The copyright holder for this preprint (which was not certified by peer review) is the author/funder. It is made available under a CC-BY 4.0 International license.

Figure S3. Cryo-EM validation and analysis of SARS-CoV-2 3Q-2P-FL spike dimers-of-trimers. (A) FSC curve for 3Q-2P-FL dimers-of-trimers map imposing C2 symmetry. **(B)** N282 glycans extending out from each trimer towards the symmetry related chain in the adjacent trimer. The adjacent spike trimers are shown in pink and blue as ribbon representation and their corresponding cryo-EM density shown in transparent gray as surface representation. **(C)** Alignment of spike sequences from representative lineage B beta-CoV strains performed using Clustal Omega. The loop residues 621-PVAIHADQ-628 are highlighted by a yellow box, the D614 residue highlighted by a blue box, the loops surrounding the NTD binding pocket are highlighted by a coral box and the potential interacting residues are underlined in black. **(D)** Surface representation of MERS spike (PDB ID: 6Q04) in tan color bound to 5-N-acetyl neuraminic acid shown in yellow. The binding site is colored in cyan. **(E)** Interaction between the protomers of adjacent trimers in the 3Q-2P-FL dimers-of-trimers model. One protomer is shown as a ribbon diagram in blue while its binding partner is shown as surface representation in gray. Residues 621-PVAIHADQ-628 on the loop with potential interactions are colored yellow and the corresponding residues in the NTD binding pocket are highlighted in coral. Spike residues predicted in glycan binding are colored in cyan.

bioRxiv preprint doi: https://doi.org/10.1101/2020.08.06.234674. this version posted August 6, 2020. The copyright holder for this preprint (which was not certified by peer review) is the author/funder. It is made available under a CC-BY 4.0 International license.

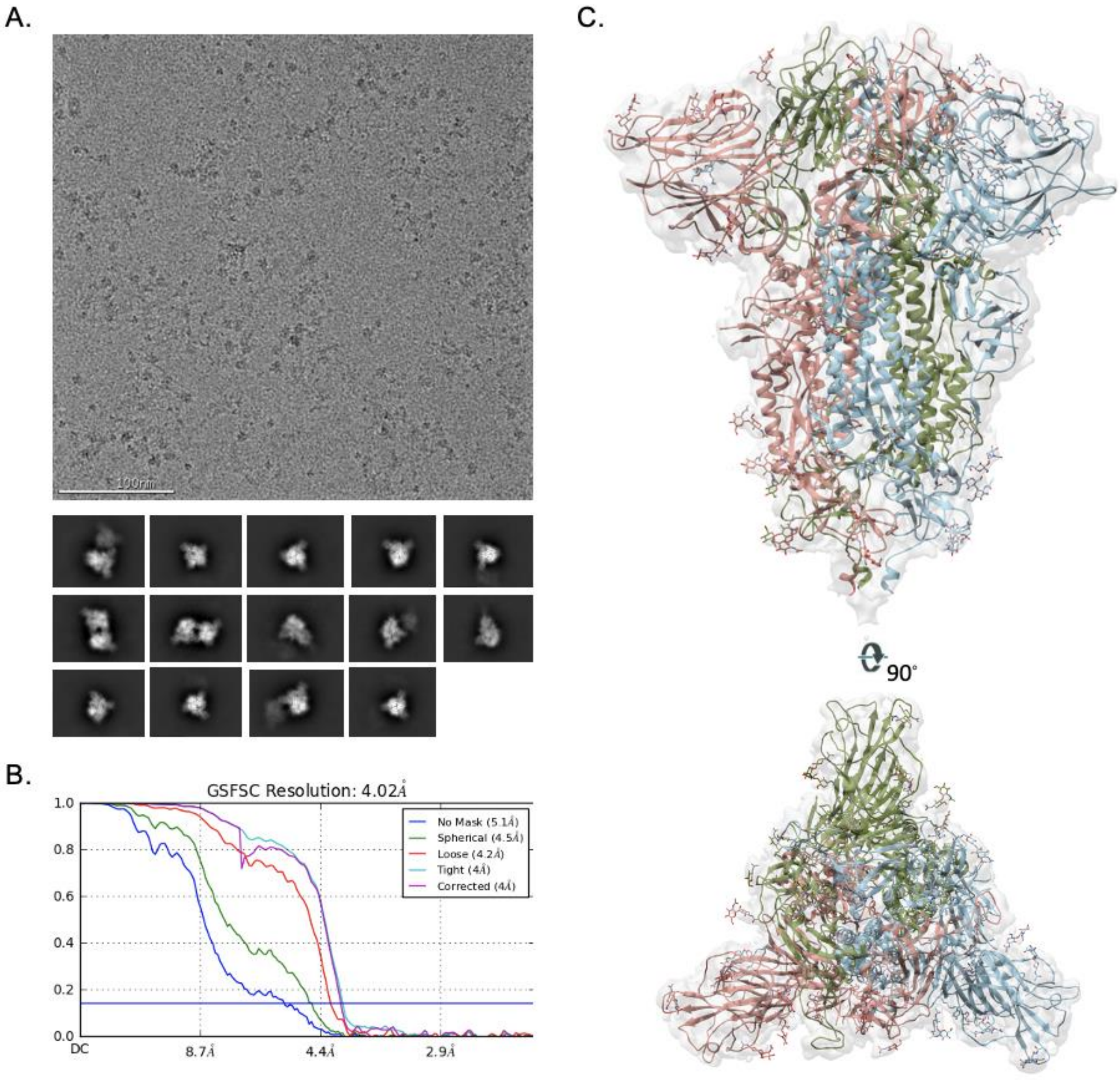

Figure S4. Cryo-EM validation and analysis of SARS-CoV-2 3Q-FL spike. (A) Representative electron micrograph and 2D class averages of 3Q-FL spikes showing free trimers and complexes of trimers. **(B)** FSC curve for SARS-CoV-2 3Q-FL spike (C3 symmetry imposed during map refinement). **(C)** Side and top view of the SARS-CoV-2 3Q-2P-FL C3 trimer atomic model represented as a ribbon diagram fit into the 3Q-FL spike C3 map density. The protomers are colored in blue, green and coral and the map is shown as a transparent gray density.

bioRxiv preprint doi: https://doi.org/10.1101/2020.08.06.234674. this version posted August 6, 2020. The copyright holder for this preprint (which was not certified by peer review) is the author/funder. It is made available under a CC-BY 4.0 International license.

Table S1. Cryo-EM data collection, refinement and model building statistics

Map	3Q-2P-FL C3 (trimer)	3Q-2P-FL C2 (dimer of trimers)	3Q-2P-FL C1 (trimer of trimer)	3Q-2P-FL C1 (trimer)	3Q-FL C3 (trimer)
EMDB	EMD-22352	EMD-22354	EMD-22355	EMD-22353	EMD-22356
**Data collection**					
Microscope	FEI Talos Arctica	FEI Talos Arctica	FEI Talos Arctica	FEI Talos Arctica	FEI Talos Arctica
Voltage (kV)	200	200	200	200	200
Detector	Gatan K2 Summit	Gatan K2 Summit	Gatan K2 Summit	Gatan K2 Summit	Gatan K2 Summit
Recording mode	Counting	Counting	Counting	Counting	Counting
Nominal magnification	36,000	36,000	36,000	36,000	36,000
Movie micrograph pixel size (Å)	1.15	1.15	1.15	1.15	1.15
Dose rate (e^-/[(camera pixel)*s])	5.7	5.7	5.7	5.7	5.95
Number of frames per movie micrograph	46	46	46	46	44
Frame exposure time (ms)	250	250	250	250	250
Movie micrograph exposure time (s)	11.5	11.5	11.5	11.5	11.1
Total dose (e^-/Å^2)	50	50	50	50	50
Defocus range (μm)	-0.5 to -1.6	-0.5 to -1.6	-0.5 to -1.6	-0.5 to -1.6	-0.4 to -2.3
**EM data processing**					
Number of movie micrographs	5,506	5,506	5,506	5,506	3,627
Number of molecular projection images in map	45,374	15,411	4,141	45,374	76,834
Symmetry	C3	C2	C1	C1	C3
Map resolution (FSC 0.143; Å)	3.6	4.5	8.0	3.8	4.0
Map sharpening B-factor (Å^2)	-114.8	-66.5	-240.3	-88.8	-123.5
**Structure Building and Validation**					
Number of atoms in deposited model					
SARS-CoV-2 S protein	25,971	52,088	N/A	N/A	N/A
Glycans	1,302	2,526	N/A	N/A	N/A
Other ligands	186	0	N/A	N/A	N/A
MolProbity score	0.76	0.75	N/A	N/A	N/A
Clashscore	0.48	0.76	N/A	N/A	N/A
Map correlation coefficient	0.85	0.80	N/A	N/A	N/A
EMRinger score	3.69	1.64	N/A	N/A	N/A
RMSD from ideal					
Bond length (Å)	0.02	0.02	N/A	N/A	N/A
Bond angles (˚)	1.74	1.79	N/A	N/A	N/A
Ramachandran plot					
Favored (%)	98.37	97.95	N/A	N/A	N/A
Allowed (%)	1.63	2.05	N/A	N/A	N/A
Outliers (%)	0.00	0.00	N/A	N/A	N/A
Side chain rotamer outliers (%)	0.21	0.07	N/A	N/A	N/A
PDB	7JJI	7JJJ	N/A	N/A	N/A
